# Postoperative Urinary Retention: A Call for Objective Assessment and Standardized Postoperative Protocols

**DOI:** 10.7759/cureus.99978

**Published:** 2025-12-23

**Authors:** Caroline Eggemann, Leandro Nobrega

**Affiliations:** 1 Obstetrics and Gynecology, Spitalzentrum Biel, Biel, CHE

**Keywords:** bladder scan, patient safety, postoperative protocols, postoperative urinary retention, post-void residual

## Abstract

Postoperative urinary retention (POUR) is a common yet under-recognized source of preventable morbidity after pelvic and gynecologic surgery. Despite its frequency and clinical impact, postoperative bladder function is frequently assessed subjectively, based on patient-reported comfort or the observation of a single “apparently normal” void, rather than through objective post-void residual (PVR) measurement. Studies using postoperative bladder ultrasound have demonstrated that clinically significant residual volumes may occur in the absence of pain or urgency, and observational data associate POUR with urinary tract infection, prolonged or repeated catheterization, impaired recovery, and an increased likelihood of subsequent intervention. Surveys further reveal substantial variation in how clinicians and institutions assess and manage postoperative bladder retention, with no consensus regarding assessment timing, PVR thresholds, or recatheterization criteria, reflecting local practice patterns rather than consolidated standards. In this editorial, we argue that POUR should be regarded as a perioperative safety domain rather than a minor inconvenience, and that standardized postoperative bladder protocols incorporating objective assessment strategies, such as routine or risk-adapted bladder ultrasound and predefined escalation pathways, represent feasible, low-cost interventions associated with improved safety and operational clarity in postoperative care workflows.

## Editorial

Postoperative urinary retention (POUR), defined as the inability to adequately empty the bladder after surgery, remains one of the most overlooked and under-evaluated yet clinically meaningful sequelae of pelvic and gynecologic procedures. Historically regarded as a transient and expected postoperative inconvenience, POUR has not received the same systematic attention as other perioperative safety domains. Enhanced recovery pathways include strict antibiotic protocols, multimodal analgesia, early ambulation, and structured venous thromboembolism prophylaxis. Yet, postoperative bladder evaluation is often left to subjective impressions, local tradition, or individual clinician judgment, despite evidence that POUR is common, predictable, and capable of generating significant morbidity if not identified early [1-4]. It is time to elevate bladder management to a defined postoperative safety priority and no longer treat it as an isolated occurrence.

Evidence confirms POUR is neither rare nor sporadic. Across a broad range of procedures, postoperative voiding dysfunction is a recurrent physiological response tied to anesthesia, perioperative fluid administration, local edema, postoperative pain, and pharmacologic effects [5,6]. In one of the largest cohorts evaluating pelvic organ prolapse repair, more than one in four patients developed POUR, with higher rates after vaginal uterosacral suspension than robotic sacrocolpopexy [1]. A recent meta-analysis of prolapse surgery reports retention rates ranging from 5% to 36%, depending on the definition used and the specific procedure performed, with age, menopause, severe cystocele, prolonged operative time, elevated preoperative residual volume, hysterectomy, and concomitant anti-incontinence procedures all increasing risk [1]. Similar retention frequencies, ranging from approximately 10% to 32%, have been reported in series involving anti-incontinence procedures and reconstructive vaginal surgery [2], while benign gynecologic surgery (such as hysterectomy or myomectomy) demonstrates lower, but still clinically relevant, POUR rates of around 3% to 4% [3]. Even in perineal tear repair, POUR may approach 20%, particularly in higher-risk settings such as severe obstetric perineal trauma, prolonged labor, operative vaginal delivery, regional anesthesia, and reoperations [4]. When a complication occurs in more than a quarter of postoperative patients undergoing common procedures, it should be regarded as foreseeable and clinically relevant rather than incidental.

One of the most serious safety gaps in current practice is the silent or subclinical nature of retention. A patient may produce an apparently adequate spontaneous void while nonetheless retaining substantial post-void residual urine capable of progressive bladder distension. In the immediate postoperative environment, the absence of discomfort does not guarantee adequate emptying. Studies using routine postoperative bladder ultrasound have demonstrated that a significant proportion of patients present large residual volumes despite no pain, urgency, or sensation of incomplete voiding [7]. In one series, nearly half of patients in the recovery room showed bladder volumes greater than 500 mL on ultrasound, and more than half of those with distension were asymptomatic and unable to void spontaneously within 30 minutes [7]. Similar findings in rehabilitation populations underscore that a meaningful proportion of patients harbor residual volumes ≥150 mL without clinical symptoms, and that systematic ultrasound screening detects cases that would otherwise be missed [8]. This phenomenon is amplified by the postoperative milieu: patients are medicated, fatigued, distracted, or reluctant to express symptoms; opioids and anticholinergics reduce detrusor contractility; neuraxial anesthesia alters bladder sensation; and perineal discomfort may inhibit spontaneous voiding [5,6]. Elderly patients or those with neurologic or pelvic floor dysfunction are especially vulnerable to silent bladder distension [6-8]. Because postoperative symptoms alone are unreliable, relying on subjective assessment invites diagnostic delay and increases risk of preventable harm [7,8].

The consequences of POUR extend far beyond transient catheter use. Bladder overdistension has been associated with urinary infection, recatheterization, impaired recovery, unplanned healthcare utilization, and increased likelihood of subsequent intervention [9-14]. Experimental and translational studies show that acute and chronic overdistension provoke hypoxia, inflammation, oxidative stress, and apoptosis within the detrusor, with the degree of injury closely linked to the duration and magnitude of overdistension [9,10]. Even a single episode of marked distension can significantly impair contractility and compliance, and functional recovery may be incomplete [9,10]. After midurethral sling placement, POUR and urinary tract infection independently predict a higher risk of surgical revision, mesh intervention, and complications [11,12]. When bladder management is handled inconsistently, the risk of infection increases through prolonged or repeated catheterization, exposing patients to avoidable antimicrobial treatment, discomfort, and delayed convalescence [12,14]. Overdistension also produces ischemic stress on detrusor smooth muscle, impairing long-term contractility and bladder compliance, particularly in elderly patients or those with pre-existing voiding impairment [9,10].

Despite well-characterized mechanisms, POUR assessment remains clinically heterogeneous. There is no standardized postoperative definition of “adequate voiding,” no universally accepted threshold for residual urine, and no agreed-upon timing for trial of void after pelvic or gynecologic surgery [5,6,13]. A large international survey of urogynecologists demonstrated striking variation in how clinicians evaluate postoperative bladder function: some require multiple spontaneous voids before discharge, others accept a single void; many do not measure residual volume routinely; thresholds for recatheterization range from 100 mL to more than 400 mL; and methods for voiding assessment vary from simple subjective impressions to structured voiding trials and ultrasound-guided strategies [13]. Only a minority of respondents reported practice informed by available evidence or locally developed institutional guidelines, underscoring the absence of consolidated standards [13]. In daily clinical settings, these discrepancies generate avoidable ambiguity for nurses, trainees, and early-career surgeons, who may be expected to make postoperative voiding decisions without institutional clarity.

This variability is not harmless. When a hospital lacks an agreed-upon algorithm, diagnostic oversight becomes likely: bladder distension may go unrecognized, patients may be recatheterized unnecessarily, or discharge decisions may be inconsistent [7,8,13]. Catheterization itself is not risk-free; it is uncomfortable, increases infection risk, disrupts ambulation, and complicates discharge planning [14]. Large database analyses and quality initiatives demonstrate that prolonged indwelling catheter use increases urinary tract infection, length of stay, and the likelihood of discharge to a non-home setting [14]. Conversely, inadequate screening for retention allows silent distension to progress until symptomatic, which may worsen bladder function and prolong hospitalization [7-10,12,14]. The inconsistency also burdens perioperative communication: nurses seek guidance from surgeons with differing preferences, residents receive conflicting instructions, and patients experience variable management depending on which clinician is on duty.

Recognizing these inconsistencies, several institutions have successfully implemented structured postoperative bladder algorithms. Quality-improvement initiatives indicate that standardized voiding assessment, with predefined bladder scan timing, residual thresholds, and escalation criteria, is associated with reduced recatheterization rates, fewer infectious complications, and improved postoperative workflow efficiency [15]. In gynecologic oncology, implementation of a standardized voiding management protocol after catheter removal has been associated with dramatic reductions in recatheterization without increasing missed retention or urinary complications [15]. Importantly, these pathways rely on simple tools: routine noninvasive bladder ultrasound, objective residual thresholds, scheduled reassessment, and clear communication among perioperative teams [7,8,15]. Such protocols do not require advanced technology, specialized urodynamics, or extended observation. Even minimalist algorithms can produce meaningful and measurable improvement when applied consistently [15].

Institutional standardization also enhances clinical clarity. When expectations are explicit, nurses can perform bladder scans and make decisions without waiting for individualized approval; surgeons have predictable postoperative parameters; anesthesiologists understand how neuraxial agents influence timing; and discharge planning becomes more consistent [13,15]. Standardization enables hospitals to monitor quality outcomes, i.e., rates of recatheterization, postoperative infections, residual thresholds, and return visits, transforming a previously invisible domain into one that is measurable and improvable [14,15]. A domain previously governed by personal preference becomes a defined patient safety process.

The ethical rationale for standardization is compelling. If core perioperative safety domains such as venous thromboembolism prophylaxis, pain management, or antibiotic stewardship are routinely standardized, postoperative bladder function warrants similar structured consideration. POUR is a foreseeable and preventable contributor to morbidity [1-4,9-12,14]. Silent retention is a diagnostic pitfall [7,8]. The cost and complexity of routine bladder scanning and simple voiding protocols are minimal compared with the downstream expense of infection, recatheterization, readmission, and functional bladder damage [9,10,14,15]. Standardized postoperative bladder protocols represent a low-cost, high-yield patient safety intervention [14,15].

National guidelines may eventually provide uniform recommendations, but institutional action does not need to wait for consensus statements. Many safety improvements in perioperative care emerged not from definitive randomized trials, but from clinical leadership willing to articulate internal priorities, align interdisciplinary expectations, and reduce ambiguity [14,15]. The absence of universal standards should not justify local variation; rather, it should motivate coordinated internal action.

POUR deserves formal recognition as a perioperative safety event requiring objective assessment rather than subjective inference [1-4,7,8]. Bladder management should be incorporated into routine pathways for pelvic and gynecologic surgery alongside catheter removal timing, pain protocols, early ambulation, and discharge milestones [3,14,15]. Clear institutional expectations, i.e., routine bladder scan after first void in at-risk populations, recatheterization above an agreed-upon residual threshold, repeat trial after a defined interval, and explicit criteria for outpatient follow-up, are simple, scalable, and clinically protective [13-15]. The priority is not a perfect international consensus on a single residual volume cutoff, but unified internal clarity.

Postoperative bladder evaluation should transition from habit-driven or varied institutional practices to standardized, evidence-based protocols. Patients deserve standardized, objective, and reliable pathways for the detection and management of urinary retention after surgery. Clinicians deserve a shared algorithm that improves workflow, minimizes ambiguity, and reduces risk. Health systems deserve traceable outcomes and reduced downstream morbidity. There is little justification for continuing to accept wide variability when structured bladder management has been shown to be feasible, inexpensive, implementable, and associated with improved safety [9,10,14,15].

## References

[1] Zhou L, Dai L, Hu S, She C, Hu P, Zhang T, Su C (2025). Risk factors for postoperative urinary retention following pelvic organ prolapse surgery: a meta-analysis. Sci Rep.

[2] Zhang BY, Wong JM, A Koenig N, Lee T, Geoffrion R (2021). Risk factors for urinary retention after urogynecologic surgery: a retrospective cohort study and prediction model. Neurourol Urodyn.

[3] Siedhoff MT, Wright KN, Misal MA, Molina AL, Greene NH (2021). Postoperative urinary retention after benign gynecologic surgery with a liberal versus strict voiding protocol. J Minim Invasive Gynecol.

[4] Kayondo M, Byamukama O, Ainomugisha B (2024). Incidence of and risk factors for post-operative urinary retention following surgery for perineal tears among Ugandan women: a prospective cohort study. Int Urogynecol J.

[5] Choi S, Mahon P, Awad IT (2012). Neuraxial anesthesia and bladder dysfunction in the perioperative period: a systematic review. Can J Anaesth.

[6] Baldini G, Bagry H, Aprikian A, Carli F (2009). Postoperative urinary retention: anesthetic and perioperative considerations. Anesthesiology.

[7] Lamonerie L, Marret E, Deleuze A, Lembert N, Dupont M, Bonnet F (2004). Prevalence of postoperative bladder distension and urinary retention detected by ultrasound measurement. Br J Anaesth.

[8] Wu J, Baguley IJ (2005). Urinary retention in a general rehabilitation unit: prevalence, clinical outcome, and the role of screening. Arch Phys Med Rehabil.

[9] Stephany HA, Strand DW, Ching CB (2013). Chronic cyclic bladder over distention up-regulates hypoxia dependent pathways. J Urol.

[10] Jin XD, Cao M, Zhou XL, Yin HP, Chen ZD, Xu N, Jiang H (2012). Effect of different durations of overdistention on rat bladder function and morphology. Braz J Med Biol Res.

[11] Ripperda CM, Kowalski JT, Chaudhry ZQ (2016). Predictors of early postoperative voiding dysfunction and other complications following a midurethral sling. Am J Obstet Gynecol.

[12] Punjani N, Winick-Ng J, Welk B (2017). Postoperative urinary retention and urinary tract infections predict midurethral sling mesh complications. Urology.

[13] Marschalek ML, Umek W, Koelbl H, Veit-Rubin N, Bodner-Adler B, Husslein H (2021). Wide variation in post-void residual management after urogynecologic surgery: a survey of urogynecologists' practices. J Clin Med.

[14] Wald HL, Ma A, Bratzler DW, Kramer AM (2008). Indwelling urinary catheter use in the postoperative period: analysis of the national surgical infection prevention project data. Arch Surg.

[15] Brackmann M, Carballo E, Uppal S, Torski J, Reynolds RK, McLean K (2020). Implementation of a standardized voiding management protocol to reduce unnecessary re-catheterization - a quality improvement project. Gynecol Oncol.

